# Genetic analysis of innate immunity in Behcet’s disease identifies an association with IL-37 and IL-18RAP

**DOI:** 10.1038/srep35802

**Published:** 2016-10-24

**Authors:** Handan Tan, Bolin Deng, Hongsong Yu, Yi Yang, Lin Ding, Qi Zhang, Jieying Qin, Aize Kijlstra, Rui Chen, Peizeng Yang

**Affiliations:** 1 The First Affiliated Hospital of Chongqing Medical University, Chongqing Key Laboratory of Ophthalmology and Chongqing Eye Institute, Chongqing, P. R. China; 2 University Eye Clinic Maastricht, Maastricht, Limburg, the Netherlands; 3 Department of Molecular and Human Genetics, Human Genome Sequencing Center, Structural and Computational Biology and Molecular Biophysics Graduate Program, The Verna and Marrs Mclean Department of Biochemistry and Molecular Biology and Program in Developmental Biology, Baylor College of Medicine, Houston, USA

## Abstract

Interleukin-1 (IL-1) and the IL-1 receptor (IL-1R) family play an important role in the pathogenesis of inflammatory diseases. This study aimed to investigate the association between single nucleotide polymorphisms (SNP) of IL-1 and IL-1R family genes with Vogt-Koyanagi-Harada (VKH) and Behcet’s disease (BD) in Han Chinese. The case-control study was divided into two stages and included 419 VKH cases, 1063 BD cases and 1872 healthy controls. The MassARRAY platform (Sequenom), iPLEX Gold Assay and TaqMan SNP assays were used to score genotypes of 24 SNPs. The expression of IL-37 and IL-18Rap was measured by ELISA and real-time PCR in genotyped healthy individuals. A significantly lower frequency of the AG genotype, and a higher frequency of the GG genotype and G allele of IL-37/rs3811047 were observed in BD as compared to controls. AA genotype and A allele frequency of IL-18RAP/rs2058660 was significantly decreased in BD as compared to controls. Functional studies performed in healthy controls showed that rs3811047 AG genotype carriers had a higher IL-37 gene expression in peripheral blood mononuclear cells (PBMCs) than GG carriers. GG carriers showed a higher cytokine expression as compared to AG carriers. No association was detected between the tested SNPs and VKH.

Uveitis is a leading cause of visual impairment and blindness, with an estimated prevalence of 38 per 100,000^1^. It can be caused by infectious or non-infectious mechanisms and classification of the disease is mainly based on the anatomical site within the eye^2^,^3^. The non-infectious uveitis entities are generally considered to be immune mediated and treatment of the disease often includes the use of corticosteroids and immunosuppressive drugs^3^,^4^. Environmental as well as hereditary factors are thought to be involved in the development of immune mediated uveitis^5^,^6^, whereby analysis of the genetic predisposition has unraveled the involvement of inflammatory pathways, leading to potential targets for treatment. Although the strongest genetic association is observed between the human leukocyte antigen (HLA) system and various uveitis entities^7^, this has not yet led to a breakthrough in the management of uveitis. Attention has therefore shifted towards analysis of genetic polymorphisms of factors that are involved in immune regulation and inflammation and various associations with uveitis have been reported in the last decade^7^,^8^,^9^,^10^,^11^,^12^,^13^.

Interaction of Interleukin-1 (IL-1) cytokine family members with their receptors are the initial steps during inflammation and dysregulated production or activity of the various members of these factors has been shown to be associated with severe immune mediated diseases like psoriatic arthritis (PsA), systemic lupus erythematosus (SLE), multiple sclerosis (MS), ankylosing spondylitis (AS), rheumatoid arthritis (RA) and type 1 diabetes (T1DM)^14^,^15^,^16^,^17^,^18^,^19^,^20^,^21^,^22^,^23^, but little is known about their role in the pathogenesis of clinical uveitis. In this study we therefore decided to investigate the association of these genes with two well defined immune mediated uveitis entities, Vogt-Koyanagi-Harada disease (VKH) and Behcet’s disease (BD)^8^. These two entities are relatively common in China, which allows the collection of large numbers of patients, providing sufficient statistical power to make reliable conclusions concerning possible associations. The IL-1 gene cluster contains 11 cytokines including IL-1α, IL-1β, IL-1Ra, IL-18, IL-33, IL-36Ra, IL-36α, IL-36β, IL- 36γ, IL-37 and IL-38^24^,^25^,^26^,^27^. The IL-1 receptor (IL-1R) family members include IL-1R1 (IL1RI), IL-1R2 (IL1RII), IL-1R3 (IL1RAP), IL-1R4 (IL1RL1), IL-1R5 (IL18R1), IL-1R6 (IL1RL2), IL-1R7 (IL18RAP), IL-1R8 (SIGIRR), IL-1R9 (IL1RAPL1), IL-1R10 (IL1RAPL2)^26^. Most of the genes of the IL-1 and IL-1R family are located in a region on chromosome 2 (2q12 and 2q13) and a coordinated regulation of these genes has been proposed^28^.

To test the association between uveitis and genetic polymorphisms in the IL-1 and IL-1R family genes, a set of single nucleotide polymorphisms (SNP) was selected. Selection was based on earlier reports on disease association, whereby linkage disequilibrium (LD) data from the Han Chinese Hap Map database were taken into account. This resulted in a selection of twenty-four SNPs, with a minor allele frequency (MAF) that was higher than 0.05 in Han Chinese. This resulted in the following ten genes: IL1A, IL1B, IL1RN, IL18, IL33, IL37, IL38, IL18RAP, IL1RL1 and IL-1 ligand cluster. Using a two stage set up we found a significant association between IL-37/rs3811047 and IL-18Rap/rs2058660 polymorphisms with BD but not with VKH.

## Results

### Clinical characteristics of VKH and BD

The genotype frequencies of the twenty-four SNPs tested, did not deviate from the Hardy-Weinberg equilibrium in healthy controls. Clinical features, age, and sex distribution in the BD, VKH and healthy controls are shown in Table 1. The clinical characteristics of the BD patients included five different features such as ulcers of the oral cavity and genital region, positive pathergy test, skin lesions, and arthritis. VKH patients had six primary features including uveitis, headache, tinnitus, alopecia, poliosis and vitiligo. The details are shown in Table 1.

### Genotype and allele frequencies of the examined SNPs in cases and controls in the first-phase study

In the first phase study, we tested the association between 24 SNPs of 10 IL-1 and IL-1R family genes with BD (n = 416) or VKH (n = 419) as compared to healthy controls (n = 627). The results demonstrated significant differences between BD cases and healthy controls for only two SNPs (rs2058660 and rs3811047) in two genes (IL-18Rap and IL-37) (Table 2). No association was observed for VKH for any of the 24 SNPs tested (Supplemental Tables 1 and 2).

Compared to controls, the frequency of the IL-37/rs3811047 AG genotype was significantly lower in BD (P_c_ = 4.32 × 10^–4^, OR = 0.522). A significant increase in the frequency of the GG genotype and G allele was observed (P_c_ = 1.66 × 10^−3^, OR = 1.800 and Pc = 2.19 × 10^−2^, OR = 1.529, respectively). The frequency of the IL-18RAP /rs2058660 AA genotype and A allele was significantly decreased in BD as compared to healthy controls (Pc = 2.08 × 10^−2^, OR = 0.603 and Pc = 1.17 × 10^−3^, OR = 0.685, respectively). The frequencies of the IL-37/rs3811047 AA genotypes and the frequencies of the IL-18RAP/rs2058660 AG and GG genotypes were not significantly different between BD cases and healthy controls (Table 2).

### Genotype and allele frequency of the examined SNPs in cases and healthy controls in the second phase and combined study

Since IL-37/rs3811047 and IL-18RAP/rs2058660 displayed a significant association with BD in the first phase exploratory study, a confirmatory second phase study was performed for these two SNPs in a separate set of BD patients (n = 647) and healthy controls (n = 1245). The data again showed that the frequency of the IL-37/rs3811047 GG genotype and G allele was significantly higher in BD as compared to controls (Pc = 3.60 × 10^−4^, OR = 1.657; P_c_ = 2.45 × 10^−5^, OR = 1.639). The AG genotype was significantly lower in BD (P_c_ = 3.25 × 10^−2^, OR = 0.672). The frequency of the AA genotype and A allele of IL-18RAP/rs2058660 was significantly decreased in BD cases as compared to controls (Pc = 3.74 × 10^−3^, OR = 0.621 and Pc = 2.51 × 10^−2^, OR = 0.788, respectively) (Table 2). Combination of the data from the first and second phase studies further strengthened the association between IL-37/rs3811047 and BD (GG genotype: Pc = 7.27 × 10^−8^, OR = 1.693; AG genotype: Pc = 3.67 × 10^−6^, OR = 0.616; G allele: Pc = 7.68 × 10^−8^, OR = 1.584) and the observed association between IL-18RAP/rs2058660 and BD (AA genotype: Pc = 1.27 × 10^−5^, OR = 0.620; A allele: Pc = 5.94 × 10^−6^, OR = 0.750) (Table 2).

### The Influence of IL/37rs3811047 and IL-18RAP/rs2058660 genotypes on mRNA and cytokine expression

To study the effect of genotype on mRNA or cytokine expression, we performed experiments with peripheral blood mononuclear cells (PBMCs) taken from healthy genotyped individuals instead of using patients. This approach was chosen in view of the ongoing inflammation in these patients and the fact that they often were receiving immunosuppressive drug therapy.

**Figure 1:**
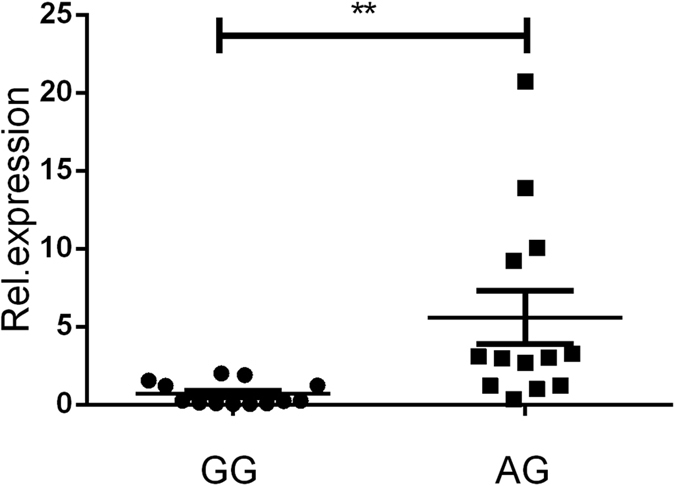
The influence of IL37/rs3811047 genotypes (GG: N = 13, AG: N = 13) on the mRNA expression of IL-37 by PBMCs obtained from healthy genotyped individuals. A statistically significant higher mRNA expression of IL-37 was observed in AG as compared to GG carriers. **P < 0.01.

**Figure 2:**
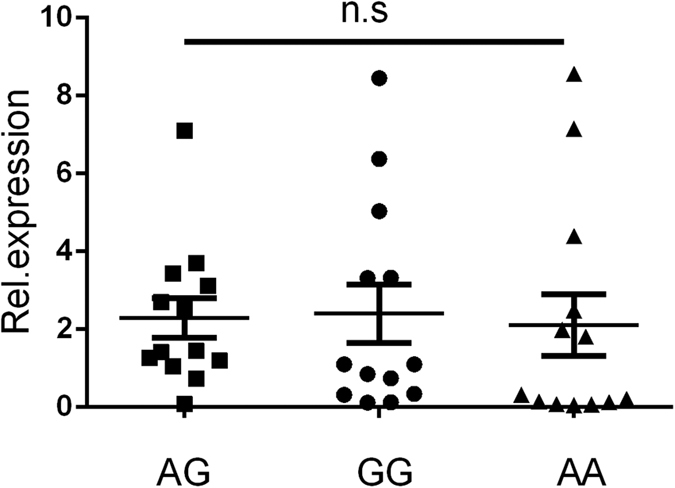
The influence of IL-18RAP/rs2058660 genotypes (GG: N = 13, AG: N = 13, AA: N = 13) on the mRNA expression of IL-18RAP in PBMCs. No statistically significant difference concerning IL-18RAP mRNA expression was detected between the different genotypes.

Real-time PCR analysis was applied to determine whether rs3811047 and rs2058660 polymorphisms altered the mRNA expression of the IL-37 or IL-18RAP gene, respectively. Functional studies using PBMCs from healthy genotyped donors indicated that rs3811047/AG carriers had a higher expression of the IL-37 gene as compared to GG carriers (Fig. 1). Polymorphisms of rs2058660 did not significantly affect the IL-18RAP expression in PBMCs from healthy individuals (Fig. 2).

**Figure 3:**
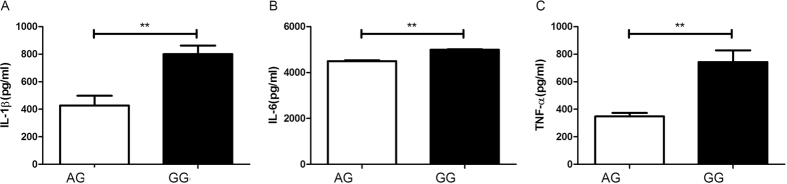
Effect of IL37/rs3811047 genotype on cytokine production by LPS stimulated PBMCs from healthy genotyped individuals (GG: N = 10, and AG: N = 10). Data are expressed as the mean ± SD. **P < 0.01.

The effect of IL-37/rs3811047 genotype on cytokine production was tested in lipopolysaccharide (LPS) stimulated PBMCs taken from genotyped healthy controls. An enzyme-linked immunosorbent assay (ELISA) was applied to examine the level of tumor necrosis factor (TNF-α), IL-1β and IL-6 in 72h cell culture supernatants. GG carriers showed a two-fold higher level of IL-1β and TNF-α as compared to that detected in AG carriers, whereas IL-6 production was only modestly increased (Fig. 3). We did not test AA carriers since the frequency of this genotype is very low (3.5%; see data of controls in Table 2).

## Discussion

This study shows that IL-37 and IL-18RAP gene polymorphisms are associated with BD but not with VKH uveitis in Han Chinese. No association could be found for gene polymorphisms in other members of the IL-1 and IL-1R family with either BD or VKH. Functional studies showed that carriers of the GG risk genotype of IL-37/rs3811047 had a higher mRNA expression of the gene and that PBMCs from such carriers showed a higher pro-inflammatory cytokine response compared to carriers of the AG genotype. An explanation for the novel association of BD with the IL-18RAP/rs2058660 gene polymorphism could not yet be supported by functional assays with PBMCs, although a recent report did show that this polymorphism was linked to a low expression of the β-chain of this factor, causing a lower response to IL-18, as detected both on the RNA and protein level of CCL3, CCL20, TNF-α and CXCL8 in granulocytes^29^. Another study that used human monocyte-derived macrophages showed that polymorphisms in this region had a marked effect on the expression of cell-surface IL-18RAP protein, leading to an altered cytokine secretion following stimulation of a wide range of pattern-recognition receptors^30^. The IL-18RAP/rs2058660 tag SNP belongs to a linkage block on chromosome 2.q12 that has a strong association with leprosy and Crohn’s disease(CD)^31^.

Our study confirms earlier reports on the association of IL-37/rs3811047 with immune mediated diseases such as PsA^14^, RA^31^ and Graves’ disease (GD)^32^. To our knowledge this is the first report addressing the association of IL-37 gene polymorphisms with uveitis. The fact that only an association was found with ocular BD and not with VKH uveitis suggests that our finding cannot be generalized for all uveitis entities and that further research is necessary to study the association of IL-37 with other uveitis types. Why only an association was found with BD and not with VKH may be caused by differences in disease causing mechanisms. BD is considered an autoinflammatory disorder caused by an aberrant response against microbial antigens, whereas VKH is mediated by a loss of immunological tolerance against melanin associated antigens. An association with factors regulating innate immunity is therefore more plausible for BD than for VKH. Earlier studies from our group however showed that IL-37 expression in PBMCs was decreased in both VKH and BD patients and that it was related to disease activity^33^,^34^. Further study in VKH patients showed that treatment with corticosteroids and cyclosporine A was associated with an increased expression of IL-37^34^. A decreased IL-37 expression in BD was confirmed recently^35^, although other groups reported that IL-37 expression was increased in immune-mediated inflammatory conditions such as RA, PsA, AS, GD and SLE^36^,^37^,^38^,^39^,^40^,^41^. The reasons for these discrepancies concerning IL-37 expression between uveitis and other autoimmune diseases is not clear and deserves further study.

IL-37 is coded by a gene that belongs to the so called IL-1 gene cluster that is located in a 360 kb region on human chromosome 2q13^42^,^43^. Other proteins coded by this region include IL-1α, IL-1β, IL-1 receptor antagonist, IL-36α, IL-36β and IL-36γ, IL-36 receptor antagonist and IL-38. We were not able to find an association between BD with other genes than IL-37 in this cluster, although others have reported an association between BD and rs16944 in the IL-1β locus^43^. The rs16944 locus is in strong linkage disequilibrium (LD) with the rs1143627 locus used in our study. The discrepancy may be due to a different BD patient subgroup, since only 54.6% of these Tunisian patients showed ocular involvement, whereas all our patients had intraocular inflammation.

There is ample evidence that IL-37 suppresses both innate as well as adaptive immune responses^44^. It is expressed in PBMCs and can be induced by Toll-like receptor agonists. IL-37 interacts with a heterologous receptor combination comprised of IL-18Ra and IL-1R8 which leads to an inhibition of the activation of inflammatory pathways in the cell^21^.

An earlier Genome-wide analysis (GWAS) in both BD and VKH did not reveal an association with IL-37 or IL-18RAP, but showed MHC class I, IL10, IL23R/IL12RB2, HLA-B*51 and ERAP1 associations with BD^45^,^46^. This may be due to the strong p value threshold used in GWAS, sample size or the use of insensitive tag SNPs. It should be noted that the observed associations were present in common variants of these genes and that the odds ratios (OR) were modest. The data are nevertheless supportive of an inflammatory pathway that involves triggering of toll like receptors (TLRs)^47^ followed by the activation of the IL-1 family. A pro-inflammatory genotype involving a myriad of cytokines and their receptors may render an individual at risk for a disease like BD, despite the fact that the OR for each individual cytokine might be low^48^.

The current study has several limitations. Although great effort was made to exactly match patients and controls for gender this was not completely achieved due to the large male preponderance in our BD group. On the other hand, we did not detect differences in genotype frequencies after stratification for gender (data not shown). Gender differences have been noted for certain geographical regions and males often suffer from the more severe forms of BD uveitis^49^. Validation of our findings is therefore necessary in other ethnic samples. All our BD patients had uveitis and it is not known whether our findings are restricted to patients with ocular BD. Further studies are therefore needed in BD patients recruited via other disciplines than ophthalmology, such as dermatology or rheumatology. A further restriction of our findings concerns the functional assays we performed. These were restricted to the testing of PBMCs, whereas recent studies suggest that addressing the response in neutrophils or macrophages might be more suitable^50^,^51^.

In conclusion, this study reports a novel association between IL-37/rs3811047 and IL-18RAP/ rs2058660 polymorphisms with BD in Han Chinese, which supports the important role of the IL-1 pathway in the pathogenesis of this disease and may provide a future target for its treatment.

## Materials and Methods

### Study Population

The study was divided into two phases: an exploratory and a confirmatory phase. A total of 3354 unrelated out-patients including 419 VKH cases, 1063 BD cases and 1872 healthy controls seen at the First Affiliated Hospital of Chongqing Medical University between April 2008 and August 2015 were included. For the first phase, 419 VKH patients and 416 BD patients as well as 627 healthy controls were tested. For the second phase, a different set of 647 BD patients and 1245 healthy controls were investigated. All subjects, either patients or controls, were Han Chinese. The controls were randomly selected among normal unrelated individuals with suitable age, ethnicity and geographic origin. Diagnostic criteria for BD and VKH disease strictly followed the International Study Group for BD and the modified diagnostic standards for VKH disease^51^,^52^,^53^. The prominent features of the BD and VKH cases in this study are shown in Table 1.

### Ethical considerations

Before the collection of blood, all the investigated individuals had signed the informed consent. The investigation protocols obtained the approval of the Clinical Research Ethics Committee of the First Affiliated Hospital of Chongqing Medical University. All experiments were conducted in accordance with the approved guidelines and regulations and the study was conducted according to the tenets of the Declaration of Helsinki.

### SNP selection and genotyping

Based on the data of Han Chinese in the HapMap database, this study utilized HaploView 4.2 to screen the candidate tag SNPs through a r2 critical value of 0.8 as well as a minor allele frequency (MAF) larger than 0.05. According to the criteria and previous reported associations of the ten IL-1 and IL-1R related genes (such as IL1A, IL1B, IL1RN, IL18, IL18RAP, IL33, IL37, IL38, IL18R1 and L1 ligand cluster) with autoimmune disease, this study selected five SNPs of IL1A (rs2071374, rs3783526, rs2856836, rs1894399, rs1800587), three SNPs of IL1B (rs1143627, rs1143643, rs2853550), five SNPs of IL1RN/ (rs1688075, rs30735, rs2234650, rs928940, rs315952), one SNP of IL18 (rs1946518), one SNP of IL18RAP (rs2058660), one SNP of IL18R1 (rs13015714), one SNP of IL37 (rs3811047), two SNPs of IL38 (rs7570267, rs3811058), four SNPs for IL33 (rs10118795, rs1929992, rs10975519, rs1048274), and one SNP of IL-1 ligand cluster (rs6712572).

All blood samples were stored in 3.2% sodium citrate-treated tubes at −80 °C, from which the DNA of BD and VKH patients and controls was extracted by utilizing the QIA amp DNA Blood Mini Kit (QIAGEN, CA). Subsequently, the collected DNA specimens were kept at −20 °C. The polymerase chain reaction (PCR) was performed on a 9700 thermocycler of the ABI Gene Amp PCR System according to the manufacturer’s instructions (Applied Biosystems, Foster City, CA). The MassARRAY platform (Sequenom, San Diego, CA) and iPLEX Gold Assay were used to score genotypes. The MassArray Designer of Sequenom was utilized to design the PCR as well as extended primers of the respective SNPs. Experimental data were analyzed through TYPER software version 4.0. In the confirmatory experiment, rs3811047 and rs2058660 genotypes were tested by the TaqMan^®^ SNP Genotyping Assay (Applied Biosystems, Foster City, CA) in the 7500 Real-Time PCR system of the Applied Biosystems. Data were analyzed with TaqMan^®^ Genotyper Software.

### Cells isolation and culture, RNA preparation and real-time quantitative PCR

Peripheral blood mononuclear cells (PBMCs) were isolated from blood obtained from healthy controls using Ficoll-Hypaque density-gradient centrifugation. TRIzol reagent (Invitrogen, Carlsbad, CA)) was used to extract RNA from PBMCs. The PrimeScript RT reagent Kit with gDNA Eraser (TaKaRa, Japan) was used to synthesize cDNA according to the manufacturer’s instructions. The Power SYBR Green PCR Master MIX (Biosystems, UK) was applied to test IL-37/IL-18RAP expression with the ABI 7500 Real-Time PCR System. The following primers of IL-37/IL-18RAP and ß-actin were used: IL-37 (forward: 5′-AGTCATCCATCCCTTCAGC-3′ and reverse: 5′-CCCAACAGGCTCATTACAA-3′), IL-18RAP (forward: 5′-CAGATATTCTGGATCCTGTCGAG-3′ and reverse: 5′-TGCTTTGCAGCTAATAGTTAAAGG-3′), β-actin (forward: 5′-CGAGAAGATGACCCAGATCATG-3′ and reverse: 5′-CAGAGGCGTACAGGGATAGCA-3′). Expressions of IL-37/IL-18RAP were calculated and compared with the expression of β-actin through the 2-ΔΔCT approach.

### Measurement of cytokines by ELISA

Human Duoset ELISA kits (R&D Systems) were used to analyze the level of IL-6, IL-1β and TNF-α in the culture supernatants of PBMCs following stimulation with LPS (100ng/ml; Sigma-Aldrich, USA) for 72 hours.

### Statistical analysis

The Chi-square (χ^2^) test was adopted to examine the Hardy-Weinberg equilibrium(HWE) in healthy samples while the genotype frequency was estimated by direct counting. The χ^2^ test was used to examine the differences between patients and healthy controls with regard to allele and genotype frequencies with SPSS17.0 statistical software package (version17.0, SPSS, Chicago, IL). Correction for multiple comparisons was performed using the Bonferroni method whereby the p value was multiplied with the number of comparisons (P corrected (P_c_)). A P_c _< 0.05, was taken as statistically significant. The Mann-Whitney test, one-way analysis of variance (ANOVA), and Kruskal-Wallis H test and Bonferroni correction were adopted by SPSS Statistics 17.0 software, where p values smaller than 0.05 were regarded as significant.

## Additional Information

**How to cite this article**: Tan, H. *et al.* Genetic analysis of innate immunity in Behcet’s disease identifies an association with IL-37 and IL-18RAP. *Sci. Rep.* **6**, 35802; doi: 10.1038/srep35802 (2016).

## Supplementary Material

Supplementary Dataset 1

Supplementary Dataset 2

## Figures and Tables

**Table 1:** Clinical features, age, and sex distribution of patients and controls.

	**Total**	**%**
**Patients with BD**	1063	
Mean age ± SD	33.9 ± 9.1	
Male	884	83.2
Female	179	16.8
Uveitis	1063	100
Oral ulcer	1063	100
Genital ulcer	590	55.5
Skin lesions	796	74.8
Arthritis	213	20.0
Positive pathergy test	171	16.1
**Patients with VKH**	419	
Mean age ± SD	39.9 ± 14.0	
Male	233	55.6
Female	186	44.4
Uveitis	419	100
Headache	176	42.0
Tinnitus	181	43.2
Alopecia	139	33.2
Vitiligo	68	16.2
Poliosis	148	35.3
**Healthy controls**	1872	
Mean age ± SD	39.4 ± 10.6	
Male	1136	60.7
Female	736	39.3

SD = standard deviation. BD = Behcet’s disease. VKH = Vogt-Koyanagi-Harada disease.

**Table 2:** Genotype and allele frequencies of IL-18Rap and IL-37 polymorphisms in BD and healthy controls.

Gene	SNPs	Stage	Genotype Allele	BD		Controls		P Value	Pc Value	OR(95% Cl)
				N	%	N	%			
IL-37	rs3811047	First	AA	13	3.1	16	2.6	0.581	NS	1.232(0.586–2.589)
			AG	92	22.1	221	35.2	6.00 × 10^–6^	4.32 × 10^−4^	0.522(0.393–0.693)
			GG	311	74.8	390	62.2	2.30 × 10^−5^	1.66 × 10^−3^	1.800(1.369–2.367)
			G	714	85.8	1001	79.8	4.57 × 10^−4^	2.19 × 10^−2^	1.529(1.205–1.942)
			A	118	14.2	253	20.2	4.57 × 10^−4^	2.19 × 10^−2^	0.654(0.515–0.830)
		Second	AA	9	1.4	50	4	0.002	NS	0.337(0.165–0.690)
			AG	141	21.8	365	29.3	4.52 × 10^−4^	3.25 × 10^−2^	0.672(0.528–0.840)
			GG	497	76.8	830	66.7	5.00 × 10^−6^	3.60 × 10^−4^	1.657(1.333–2.059)
			G	1135	87.7	2025	81.3	5.10 × 10^−7^	2.45 × 10^−5^	1.639(1.350–1.990)
			A	159	12.3	465	18.7	5.10 × 10^−7^	2.45 × 10^−5^	0.610(0.502–0.741)
		Combined	AA	22	2.1	66	3.5	0.026	NS	0.578(0.355–0.943)
			AG	233	21.9	586	31.3	5.10 × 10^−8^	3.67 × 10^−6^	0.616(0.517–0.734)
			GG	808	76	1220	65.2	1.01 × 10^−9^	7.27 × 10^−8^	1.693(1429–2.007)
			G	1849	87	3026	80.8	1.60 × 10^−9^	7.68 × 10^−8^	1.584(1.363–1.841)
			A	277	13	718	19.2	1.60 × 10^−9^	7.68 × 10^−8^	0.631(0.543–0.734)
IL-18RAP	rs2058660	First	AA	89	21.4	195	31.1	5.64 × 10^−4^	2.08 × 10^−2^	0.603(0.452–0.805)
			AG	194	46.6	289	46.1	0.863	NS	1.022(0.797–1.310)
			GG	133	32	143	22.8	0.001	NS	1.591(1.205–2.100)
			A	372	44.7	679	54.1	2.44 × 10^−5^	1.17 × 10^−3^	0.685(0.574–0.817)
			G	460	55.3	575	45.9	2.44 × 10^−5^	1.17 × 10^−3^	1.460(1.224–1.741)
		Second	AA	124	19.2	344	27.6	5.20 × 10^−5^	3.74 × 10^−3^	0.621(0.492–0.783)
			AG	346	53.5	603	48.4	0.037	NS	1.224(1.012–1.480)
			GG	177	27.3	298	24.0	0.104	NS	1.197(0.964–1.486)
			A	594	45.9	1291	51.8	5.23 × 10^−4^	2.51 × 10^−2^	0.788(0.689–0.902)
			G	700	54.1	1199	48.2	5.23 × 10^−4^	2.51 × 10^−2^	1.269(1.109–1.452)
		Combined	AA	213	20	539	28.8	1.77 × 10^−7^	1.27 × 10^−5^	0.620(0.517–0.742)
			AG	540	50.8	892	47.6	0.101	NS	1.134(0.976–1.319)
			GG	310	29.2	441	23.6	8.24 × 10^−4^	NS	1.336(1.127–1.583)
			A	966	45.4	1970	52.6	1.24 × 10^−7^	5.94 × 10^−6^	0.750(0.674–0.834)
			G	1160	54.6	1774	47.4	1.24 × 10^−7^	5.94 × 10^−6^	1.334(1.198–1.484)

SNP = single-nucleotide polymorphism; BD = 5% confidence interval; Pc = Bonferroni corrected p value.

## References

[b1] Rothova A., Schulten M. S., Treffers W. & Kijlstra A. Causes and frequency of blindness in patients with intraocular inflammatory disease. Br J Ophthalmol 80, 332–336 (1996). 8703885 10.1136/bjo.80.4.332 PMC505460

[b2] Hassan A., Al-Dhibi, Ammar M. & Al-Mahmood Arevalo J. F. A systematic approach to emergencies in uveitis. Middle East Afr J Ophthalmol 21, 251–258 (2014). 25100911 10.4103/0974-9233.134687 PMC4123279

[b3] Caspi R. R. A look at autoimmunity and inflammation in the eye. J Clin Invest 120, 3073–3083 (2010). 20811163 10.1172/JCI42440 PMC2929721

[b4] Moen E. L., Godley L. A., Zhang W. & Dolan M. E. Pharmacogenomics of chemotherapeutic susceptibility and toxicity. Genome Med 4, 2791–2800 (2012). 10.1186/gm391 PMC3580423 23199206

[b5] Yang P. *et al.* Clinical characteristics of Vogt-Koyanagi-Harada syndrome in Chinese patients. Ophthal mology 114, 606–614 (2007). 10.1016/j.ophtha.2006.07.040 17123618

[b6] Murthy S. I. *et al.* The spectrum of Vogt-Koyanagi-Harada disease in South India. Int Ophthalmol 27, 131–136 (2007). 17318321 10.1007/s10792-007-9046-9

[b7] Hou S., Kijlstra A. & Yang P. Molecular Genetic Advances in Uveitis. Prog Mol Biol Transl Sci 134, 283–298 (2015). 26310161 10.1016/bs.pmbts.2015.04.009

[b8] Hou S. *et al.* Genetic variations of IL17F and IL23A show associations with Behcet’s disease and Vogt-Koyanagi-Harada syndrome. Ophthalmology 122, 518–523 (2015). 25439430 10.1016/j.ophtha.2014.09.025

[b9] Yu H. *et al.* FAS Gene Copy Numbers are Associated with Susceptibility to Behçet Disease and VKH Syndrome in Han Chinese. Hum Mutat 36, 1064–1069 (2015). 26136352 10.1002/humu.22829

[b10] Yu H., Liu Y., Bai L., Kijlstra A. & Yang P. Predisposition to Behçet’s disease and VKH syndrome by genetic variants of miR-182. J Mol Med (Berl) 92, 961–967 (2014). 24801147 10.1007/s00109-014-1159-9

[b11] Li L. *et al.* Genetic Variations of NLR family genes in Behcet’s Disease. Sci Rep 6, 20098 (2016). 26833430 10.1038/srep20098 PMC4735577

[b12] Jiang Y. *et al.* Two Genetic Variations in the IRF8 region are associated with Behçet’s disease in Han Chinese. Sci Rep 6, 19651 (2016). 26794091 10.1038/srep19651 PMC4726413

[b13] Yu H. *et al.* Identification of susceptibility SNPs in IL10 and IL23R-IL12RB2for Behçet’s disease in Han Chinese. J Allergy Clin Immunol, doi: 10.1016/j.jaci.2016.05.024 (2016) 27464962

[b14] Ge R. *et al.* Analysis on the interaction between IL-1F7 gene and environmental factors on patients with ankylosing spondylitis: a case-only study. Mol Biol Rep 38, 2281–2284 (2011). 21072596 10.1007/s11033-010-0359-9

[b15] Rahman P. *et al.* Association between the interleukin-1 family gene cluster and psoriatic arthritis. Arthritis Rheum 54, 2321–2325 (2006). 16918024 10.1002/art.21928

[b16] Guo Z. S. *et al.* Association of IL-1 gene complex members with ankylosing spondylitis in Chinese Han population. Int J Immuno genet 37, 33–37 (2010). 10.1111/j.1744-313X.2009.00889.x 19930406

[b17] Maksymowych W. P. *et al.* Association of the IL1 gene cluster with susceptibility to ankylosing spondylitis: an analysis of three Canadian populations. Arthritis Rheum 54, 974–985 (2006). 16508980 10.1002/art.21642

[b18] Latiano A., Palmieri O., Pastorelli L., Vecchi M., Pizarro T. T. & Bossa F. Associations between genetic polymorphisms in IL-33, IL1R1 and risk for inflammatory bowel disease. PLoS One 8, e62144 (2013). 23634226 10.1371/journal.pone.0062144 PMC3636262

[b19] Yamamoto-Furusho J. K. *et al.* Interleukin 1 beta (IL-1B) and IL-1 antagonist receptor (IL-1RN) gene polymorphisms are associated with the genetic susceptibility and steroid dependence in patients with ulcerative colitis. J Clin Gastroenterol 45, 531–535 (2011). 20975573 10.1097/MCG.0b013e3181faec51

[b20] Harrison P. *et al.* Interleukin-1 promoter region polymorphism role in rheumatoid arthritis: a meta-analysis of IL-1B-511A/G variant reveals association with rheumatoid arthritis. Rheumatology (Oxford) 47, 1768–1770 (2008). 18838388 10.1093/rheumatology/ken374

[b21] Tahmasebi Z. *et al.* Interleukin-1 gene cluster and IL-1 receptor polymorphisms in Iranian patients with systemic lupus erythemato- sus. Rheumatol Int 33, 2591–2596 (2013). 23722873 10.1007/s00296-013-2784-2

[b22] Sarial S. *et al.* IL-1, IL-1R and TNF α Gene Polymorphisms in Iranian Patients with Multiple Sclerosis. Iran J Allergy Asthma Immunol 7, 37–40 (2008). 18322311

[b23] Tavares N. A. *et al.* Interleukin 18 (IL18) gene promoter polymorphisms are associated with type 1 diabetes mellitus in Brazilian patients. Cytokine 62, 286–289 (2013). 23557801 10.1016/j.cyto.2013.03.004

[b24] Dinarello C. *et al.* IL-1 family nomenclature. Nat Immunol 11, 973 (2010) 20959797 10.1038/ni1110-973 PMC4174560

[b25] Garlanda C., Dinarello C. A. & Mantovani C. A. The interleukin-1 family: back to the future. Immun-ity 39, 1003–1018 (2013). 10.1016/j.immuni.2013.11.010 PMC3933951 24332029

[b26] Boraschi D. & Tagliabue A. The interleukin-1 receptor family. Semin Immunol 25, 394–407 (2013). 24246227 10.1016/j.smim.2013.10.023

[b27] Aggarwal A. *et al.* IL1 gene polymorphisms in enthesitis related arthritis category of juvenile idiopathic arthritis (ERA-JIA). Clin Rheumatol 31, 607–611 (2012). 22124790 10.1007/s10067-011-1898-8

[b28] Sharaf N., Nicklin M. J. & Giovine F. S. Long-range DNA interactions at the IL-1/IL-36/IL-37 gene cluster (2q13) are induced by activation of monocytes. Cytokine 68, 16–22 (2014). 24787052 10.1016/j.cyto.2014.03.002

[b29] Andiappan A. K. *et al.* Genome-wide analysis of the genetic regulation of gene expression in human neutrophils. Nat Commun 6, 7971 (2015). 26259071 10.1038/ncomms8971 PMC4918343

[b30] Hedl M., Zheng S. & Abraham C. The IL18RAP region disease polymorphism decreases IL-18RAP/IL-18R1/IL-1R1 expression and signaling through innate receptor-initiated pathways. J Immunol 192, 5924–5932 (2014). 24842757 10.4049/jimmunol.1302727 PMC4146459

[b31] Pei B. *et al.* Associations of the IL-1F7 gene polymorphisms with rheumatoid arthritis in Chinese Han population. Int J Immunoge- net 40, 199–203 (2013). 10.1111/iji.12007 23171316

[b32] Yan N. *et al.* Polymorphism of IL37 gene as a protective factor for autoimmune thyroid disease. J Mol Endocrinol 55, 209–218 (2015). 26373794 10.1530/JME-15-0144

[b33] Ye Z. *et al.* A possible role for interleukin 37 in the pathogenesis of Behcet’s disease. Curr Mol Med 14, 535–542 (2014). 24730521 10.2174/1566524014666140414210831

[b34] Ye Z. *et al.* Decreased interleukin-37 expression in Vogt-Koyanagi-Harada disease and upregulation following immunosuppress sive treatment. J Interferon Cytokine Res 35, 265–272 (2015). 25343528 10.1089/jir.2014.0042

[b35] Bouali E., Kaabachi W., Hamzaoui A. & Hamzaoui K. Interleukin-37 expression is decreased in Behcet’s disease and is associated with inflammation. Immunol Lett 167, 87–94 (2015). 26253248 10.1016/j.imlet.2015.08.001

[b36] Zhao P. W. *et al.* Plasma levels of IL-37 and correlation with TNF-alpha, IL-17A, and disease activity during DMARD treatment of rheumatoid arthritis. PLoS One 9, e95346 (2014). 24788826 10.1371/journal.pone.0095346 PMC4006923

[b37] Ye L. *et al.* IL-37 Alleviates Rheumatoid Arthritis by Suppressing IL-17 and IL-17-Triggering Cytokine Production and Limiting Th17 Cell Proliferation. J Immunol 194, 5110–5119 (2015). 25917106 10.4049/jimmunol.1401810

[b38] Teng X. *et al.* IL-37 ameliorates the inflammatory process in psoriasis by suppressing proinflammatory cytokine production. J Im munol 192, 1815–1823 (2014). 10.4049/jimmunol.1300047 24453242

[b39] Chen B. *et al.* Interleukin-37 is increased in ankylosing spondylitis patients and associated with disease activity. J Transl Med 13, 36 (2015). 25627863 10.1186/s12967-015-0394-3 PMC4323018

[b40] Li Y. *et al.* Increased expression of IL-37 in patients with Graves’ disease and its contribution to suppression of proinflammatory cytokines production in peripheral blood mononuclear cells. PLoS One 9, e107183 (2014). 25226272 10.1371/journal.pone.0107183 PMC4165889

[b41] Ye L. *et al.* IL-37 inhibits the production of inflammatory cytokines in peripheral blood mononuclear cells of patients with systemic lupus erythematosus: its correlation with disease activity. J Transl Med 12, 69 (2014). 24629023 10.1186/1479-5876-12-69 PMC4003851

[b42] Nicklin M. J. *et al.* A sequence-based map of the nine genes of the human interleukin-1 cluster. Genomics 79, 718–725 (2002). 11991722 10.1006/geno.2002.6751

[b43] Ozcimen A. A. *et al.* IL-1 cluster gene polymorphisms in Turkish patients with Behcet’s disease. Int J Immunogenet 38, 295–301 (2011). 21418526 10.1111/j.1744-313X.2011.01006.x

[b44] Xu W. D., Zhao Y. & Liu Y. Insights into IL-37, the role in autoimmune diseases. Autoimmun Rev 14, 1170–1175 (2015). 26264940 10.1016/j.autrev.2015.08.006

[b45] Remmers E. F. *et al.* Genome-wide association study identifies variants in the MHC class I, IL10, and IL23R/IL12RB2 regions associated with Behçet’s disease. Nat Genet 42, 698–702 (2010). 20622878 10.1038/ng.625 PMC2923807

[b46] Yohei-Kirino. *et al.* Genome-wide association analysis identifies new susceptibility loci for Behçet’s disease and epistasis between HLA-B*51 and ERAP1. Nat Genet 45, 202–207 (2013). 23291587 10.1038/ng.2520 PMC3810947

[b47] Liu X. *et al.* Higher expression of Toll-like receptors 2, 3, 4, and 8 in ocular Behcet’s disease. Invest Ophthalmol Vis Sci 54, 6012–6017 (2013). 23908180 10.1167/iovs.13-12159

[b48] Morton L. T. *et al.* Genetics of Behçet’s disease. Curr Opin Rheumatol 28, 39–44 (2016). 26599381 10.1097/BOR.0000000000000234

[b49] Ucar-Comlekoglu D. *et al.* Gender Differences in Behçet’s Disease Associated Uveitis. J Ophthalmol 2014, 820710 (2014). 24864195 10.1155/2014/820710 PMC4017716

[b50] Majai G. *et al.* Decreased apopto-phagocytic gene expression in the macrophages of systemic lupus erythematosus patients. Lupus. 23, 133–145 (2014). 24285095 10.1177/0961203313511557

[b51] Bossaller L. *et al.* Overexpression of membrane-bound fas ligand (CD95L) exacerbates autoimmune disease and renal pathology in pristane-induced lupus. J Immunol. 191, 2104–2114 (2013). 23918976 10.4049/jimmunol.1300341 PMC4219920

[b52] Listed N. A. Criteria for diagnosis of Behçet’s disease. International Study Group for Behçet’s Disease. Lancet 335, 1078–1080 (1990). 1970380

[b53] Read R. W. *et al.* Revised diagnostic criteria for Vogt-Koyanagi-Harada disease: report of an international committee on nomencla- ture. Am J Ophthalmol 131, 647–652 (2001). 11336942 10.1016/s0002-9394(01)00925-4

