# Supplementary material for: Genetic analysis of innate immunity in Behcet’s disease identifies an association with IL-37 and IL-18RAP
Source: Sci Rep. 2016 Oct 24;6:35802. doi: 10.1038/srep35802 (PMC5075872; doi:10.1038/srep35802)
Supplement: Supplementary Dataset 2 [file srep35802-s2.doc]

**Genetic analysis of innate immunity in Behcet’s disease identifies an**

**association with IL-37 and IL-18RAP**

**Handan Tan^1#^, Bolin Deng^1#^, Hongsong Yu^1^, Yi Yang^1^, Lin Ding^1^, Qi Zhang^1^, Jieying Qin^1^, Aize Kijlstra^2^, Rui Chen^3^, Peizeng Yang^1*^**

1 The First Affiliated Hospital of Chongqing Medical University, Chongqing Key Laboratory of Ophthalmology and Chongqing Eye Institute, Chongqing, P. R. China

2 University Eye Clinic Maastricht, Maastricht, Limburg, the Netherlands

3 Department of Molecular and Human Genetics, Human Genome Sequencing Center, Structural and Computational Biology and Molecular Biophysics Graduate Program, The Verna and Marrs Mclean Department of Biochemistry and Molecular Biology and Program in Developmental Biology, Baylor College of Medicine, Houston, USA

**#**These authors contributed equally to this work.

**Supplemental Table 2 The frequency of genotypes and alleles of IL-1 and IL-1R family genes except IL-18Rap in VKH versus healthy controls.**

| Gene | SNPs | Genotype | Control | VKH | P value | Pc value | OR(95%CI) |
| --- | --- | --- | --- | --- | --- | --- | --- |
|  |  | Allele | n(%) | n(%) |  |  |  |
| IL1A | rs2071374 | GG | 22(3.5) | 19(4.6) | 0.648 | NS | 1.158(0.617-2.173) |
|  |  | GT | 210(33.4) | 148(35.6) | 0.476 | NS | 0.910(0.701-1.180) |
|  |  | TT | 396(63.1) | 249(59.9) | 0.297 | NS | 1.145(0.888-1.476) |
|  |  | G | 254(20.2) | 186(22.4) | 0.242 | NS | 1.136(0.918-1.406) |
|  |  | T | 1002(79.8) | 646(77.6) | 0.242 | NS | 0.880(0.711-1.090) |
|  | rs3783526 | CC | 71(11.8) | 44(10.9) | 0.854 | NS | 1.030(0.696-1.549) |
|  |  | CT | 256(42.6) | 188(46.4) | 0.055 | NS | 0.799(0.601-1.005) |
|  |  | TT | 274(45.6) | 153(57.3) | 0.070 | NS | 1.271(0.980-1.647) |
|  |  | C | 398(33.1) | 276(35.8) | 0.212 | NS | 1.129(0.933-1.365) |
|  |  | T | 804(66.9) | 494(64.2) | 0.212 | NS | 0.886(0.733-1.071) |
|  | rs2856836 | GG | 7(1.1) | 4(1.0) | 1 | NS | 1.084(0.315-3.729) |
|  |  | GA | 98(15.6) | 67(17.5) | 0.493 | NS | 0.888(0.631-1.248) |
|  |  | AA | 512(83.0) | 311(81.4) | 0.527 | NS | 1.113(0.798-1.552) |
|  |  | A | 1122(90.9) | 689(90.2) | 0.581 | NS | 0.917(0.674-1.247) |
|  |  | G | 112(9.1) | 75(9.8) | 0.581 | NS | 1.090(0.802-1.483) |
|  | rs1894399 | TT | 6(1.0) | 8(2.0) | 0.159 | NS | 0.473(0.163-1.374) |
|  |  | TC | 110(17.8) | 58(14.7) | 0.199 | NS | 1.254(0.887-1.774) |
|  |  | CC | 502(81.2) | 328(83.2) | 0.415 | NS | 0.871(0.624-1.215) |
|  |  | C | 1114(90.1) | 714(90.6) | 0.722 | NS | 1.057(0.780-1.432) |
|  |  | T | 122(9.9) | 74(9.4) | 0.722 | NS | 0.946(0.699-1.282) |
|  | rs1800587 | GG | 514(82.8) | 332(81.8) | 0.682 | NS | 1.071(0.772-1.485) |
|  |  | GA | 100(16.1) | 70(17.2) | 0.631 | NS | 0.921(0.659-1.288) |
|  |  | AA | 7(1.1) | 4(1.0) | 0.829 | NS | 1.146(0.333-3.939) |
|  |  | A | 114(9.2) | 78(9.6) | 0.745 | NS | 1.051(0.777-1.423) |
|  |  | G | 1128(90.8) | 734(90.4) | 0.745 | NS | 0.951(0.703-1.287) |
| IL1B | rs1143643 | CC | 148(24.6) | 103(25.1) | 0.965 | NS | 0.994(0.743-1.329) |
|  |  | CT | 284(48.8) | 193(47.0) | 0.779 | NS | 1.037(0.806-1.334) |
|  |  | TT | 160(26.6) | 114(27.7) | 0.786 | NS | 0.962(0.725-1.275) |
|  |  | C | 580(49.0) | 399(48.7) | 0.885 | NS | 0.987(0.826-1.179) |
|  |  | T | 604(51.0) | 421(51.3) | 0.885 | NS | 1.013(0.848-1.211) |
|  | rs1143627 | AA | 146(24.8) | 118(29.1) | 0.139 | NS | 0.807(0.607-1.073) |
|  |  | AG | 294(49.9) | 188(46.3) | 0.248 | NS | 1.161(0.901-1.495) |
|  |  | GG | 149(25.3) | 101(24.9) | 0.863 | NS | 1.026(0.766-1.373) |
|  |  | A | 586(49.7) | 424(52.1) | 0.304 | NS | 1.098(0.919-1.313) |
|  |  | G | 592(50.3) | 390(47.9) | 0.304 | NS | 0.910(0.761-1.089) |
|  | rs2853550 | GG | 482(77.7) | 305(81.3) | 0.177 | NS | 1.247(0.905-1.720) |
|  |  | GA | 130(21.0) | 60(16.0) | 0.053 | NS | 0.718(0.513-1.006) |
|  |  | AA | 8(1.3) | 10(2.7) | 0.605 | NS | 1.281(0.500-3.282) |
|  |  | A | 146(11.8) | 80(10.7) | 0.45 | NS | 0.895(0.670-1.194) |
|  |  | G | 1094(88.2) | 670(89.3) | 0.45 | NS | 1.118(0.837-1.492) |
| IL1RN | rs2234650 | TT | 34(5.4) | 26(6.2) | 0.57 | NS | 1.165(0.688-1.972) |
|  |  | TC | 220(35.0) | 150(36.1) | 0.734 | NS | 1.046(0.807-1.355) |
|  |  | CC | 374(59.6) | 240(57.7) | 0.55 | NS | 0.926(0.720-1.191) |
|  |  | C | 968(77.1) | 630(75.7) | 0.476 | NS | 0.928(0.755-1.140) |
|  |  | T | 288(22.9) | 202(24.3) | 0.476 | NS | 1.078(0.877-1.324) |
|  | rs928940 | GG | 155(25.6) | 92(24.1) | 0.613 | NS | 1.080(0.802-1.453) |
|  |  | GT | 296(48.8) | 186(48.8) | 0.994 | NS | 1.001(0.775-1.294) |
|  |  | TT | 155(25.6) | 103(27.0) | 0.612 | NS | 0.928(0.694-1.240) |
|  |  | G | 606(50.0) | 370(48.6) | 0.532 | NS | 0.944(0.787-1.131) |
|  |  | T | 606(50.0) | 392(51.4) | 0.532 | NS | 1.059(0.884-1.270) |
|  | rs315952 | CC | 222(36.6) | 130(33.4) | 0.31 | NS | 1.149(0.879-1.501) |
|  |  | CT | 294(48.4) | 193(49.6) | 0.716 | NS | 0.954(0.739-1.231) |
|  |  | TT | 91(15.0) | 66(17.0) | 0.404 | NS | 0.863(.611-1.220) |
|  |  | C | 738(60.8) | 453(58.2) | 0.255 | NS | 0.899(0.748-1.080) |
|  |  | T | 476(39.2) | 325(41.8) | 0.255 | NS | 1.112(0.926-1.336) |
|  | rs1688075 | CC | 175(29.8) | 124(31.2) | 0.640 | NS | 0.936(0.710-1.234) |
|  |  | CA | 287(48.9) | 192(48.2) | 0.861 | NS | 1.023(0.793-1.320) |
|  |  | AA | 126(21.5) | 82(20.6) | 0.755 | NS | 1.053(0.769-1.437) |
|  |  | A | 539(45.8) | 356(44.7) | 0.627 | NS | 0.956(0.798-1.146) |
|  |  | C | 637(54.2) | 440(55.3) | 0.627 | NS | 1.046(0.873-1.253) |
|  | rs30735 | GG | 216(32.5) | 120(30.5) | 0.121 | NS | 1.239(0.945-1.625) |
|  |  | GA | 288(46.9) | 195(49.5) | 0.422 | NS | .902(0.700-1.161) |
|  |  | AA | 110(17.9) | 79(20.1) | 0.397 | NS | 0.870(0.631-1.200) |
|  |  | A | 508(41.4) | 353(44.8) | 0.129 | NS | 1.150(0.960-1.378) |
|  |  | G | 720(58.6) | 435(55.2) | 0.129 | NS | 0.869(0.726-1.042) |
| IL18 | rs1946518 | GG | 145(24.5) | 87(21.9) | 0.356 | NS | 1.153(0.852-1.561) |
|  |  | GT | 291(49.1) | 215(54.2) | 0.117 | NS | 0.816(0.632-1.052) |
|  |  | TT | 157(26.5) | 95(23.9) | 0.367 | NS | 1.145(0.853-1.536) |
|  |  | G | 581(49.0) | 389(49.0) | 0.999 | NS | 1.000(0.836-1.197) |
|  |  | T | 605(51.0) | 405(51.0) | 0.999 | NS | 1.000(0.836-1.197) |
| IL18R1 | rs13015714 | GG | 142(24.1) | 107(26.8) | 0.328 | NS | 1.136(0.864-1.547) |
|  |  | GT | 278(47.1) | 208(52.1) | 0.122 | NS | 1.222(0.948-1.576) |
|  |  | TT | 170(28.8) | 84(21.1) | 0.006 | NS | 0.659(0.488-0.889) |
|  |  | G | 562(47.6) | 422(52.9) | 0.0219 | NS | 1.234(1.0310-1.477) |
|  |  | T | 618(52.4) | 376(47.1) | 0.0219 | NS | 0.810(0.677-0.970) |
| IL-38 | rs7570267 | AA | 456(79.4) | 314(75.1) | 0.107 | NS | 1.280(0.948-1.728) |
|  |  | AG | 107(18.6) | 93(22.2) | 0.162 | NS | 0.801(0.586-1.094) |
|  |  | GG | 11(1.9) | 11(2.7) | 0.45 | NS | 0.723(0.310-1.684) |
|  |  | A | 1019(88.8) | 721(86.2) | 0.092 | NS | 0.794(0.607-1.039) |
|  |  | G | 129(11.2) | 115(13.8) | 0.092 | NS | 1.260(0.963-1.649) |
|  | rs3811058 | CC | 196(32.1) | 139(33.3) | 0.667 | NS | 0.943(0.724-1.230) |
|  |  | CT | 301(49.3) | 196(47.0) | 0.483 | NS | 1.093(0.852-1.403) |
|  |  | TT | 113(18.5) | 81(19.4) | 0.704 | NS | 0.940(0.685-1.291) |
|  |  | C | 693(56.8) | 474(57.0) | 0.940 | NS | 1.007(0.843-1.203) |
|  |  | T | 527(43.2) | 358(43.0) | 0.940 | NS | 0.993(0.831-1.187) |
| IL33 | rs10118795 | TT | 121(19.7) | 69(17.0) | 0.274 | NS | 1.200(0.866-1.663) |
|  |  | TC | 309(50.2) | 215(52.8) | 0.419 | NS | 0.902(0.702-1.159) |
|  |  | CC | 185(30.1) | 123(30.2) | 0.962 | NS | 0.993(0.756-1.305) |
|  |  | C | 679(55.2) | 461(56.6) | 0.524 | NS | 1.060(0.887-1.267) |
|  |  | T | 551(44.8) | 353(43.4) | 0.524 | NS | 0.944(0.789-1.128) |
|  | rs1929992 | CC | 168(27.3) | 119(29.4) | 0.463 | NS | 0.901(0.683-1.190) |
|  |  | CT | 326(52.9) | 211(52.1) | 0.797 | NS | 1.034(0.804-1.329) |
|  |  | TT | 122(19.8) | 75(18.5) | 0.61 | NS | 1.087(0.789-1.496) |
|  |  | C | 662(53.7) | 449(55.4) | 0.451 | NS | 1.071(0.896-1.280) |
|  |  | T | 570(46.3) | 361(44.6) | 0.451 | NS | 0.934(0.781-1.116) |
|  | rs10975519 | CC | 163(27.0) | 85(12.9) | 0.066 | NS | 1.325(0.981-1.789) |
|  |  | CT | 273(43.5) | 200(51.4) | 0.059 | NS | 0.782(0.606-1.009) |
|  |  | TT | 167(27.7) | 104(26.7) | 0.741 | NS | 1.050(0.788-1.398) |
|  |  | C | 599(49.7) | 370(47.6) | 0.359 | NS | 0.919(0.767-1.101) |
|  |  | T | 607(50.3) | 408(52.4) | 0.359 | NS | 1.088(0.909-1.303) |
|  | rs1048274 | AA | 160(26.1) | 105(25.7) | 0.908 | NS | 1.017(0.764-1.354) |
|  |  | AG | 310(50.5) | 197(48.3) | 0.49 | NS | 1.092(0.850-1.403) |
|  |  | GG | 144(23.5) | 106(26.0) | 0.357 | NS | 0.873(0.653-1.168) |
|  |  | A | 630(51.3) | 407(49.9) | 0.528 | NS | 0.945(0.791-1.128) |
|  |  | G | 598(48.7) | 409(50.1) | 0.528 | NS | 1.059(0.887-1.264) |
| IL-1 ligand cluster | rs6712572 | GG | 250(42.4) | 156(39.4) | 0.34 | NS | 1.135(0.875-1.471) |
|  |  | GT | 264(44.8) | 197(49.7) | 0.129 | NS | 0.821(0.636-1.059) |
|  |  | TT | 75(12.7) | 43(10.9) | 0.374 | NS | 1.198(0.804-1.785) |
|  |  | G | 764(64.9) | 509(64.3) | 0.789 | NS | 0.975(0.807-1.176) |
|  |  | T | 414(35.1) | 283(35.7) | 0.789 | NS | 1.026(0.850-1.239) |
| IL-37 | rs3811047 | AA | 16(2.6) | 5(1.2) | 0.125 | NS | 0.461(0.168-1.269) |
|  |  | AG | 221(35.2) | 185(44.2) | 0.888 | NS | 1.018(0.798-1.298) |
|  |  | GG | 390(62.2) | 229(54.7) | 0.015 | NS | 0.732(0.570-0.941) |
|  |  | G | 1001(79.8) | 195(23.3) | 0.091 | NS | 0.833(0.675-1.030) |
|  |  | A | 253(20.2) | 643(76.7) | 0.091 | NS | 1.200(0.971-1.482) |
| IL-18RAP | rs2058660 | AA | 195(31.1) | 139(33.2) | 0.481 | NS | 1.100(0.844-1.433) |
|  |  | AG | 289(46.1) | 169(40.3) | 0.066 | NS | 0.791(0.615-1.016) |
|  |  | GG | 143(22.8) | 111(26.5) | 0.173 | NS | 1.220(0.916-1.624) |
|  |  | A | 679(54.1) | 447(53.3) | 0.717 | NS | 0.968(0.812-1.154) |
|  |  | G | 575(45.9) | 391(46.7) | 0.717 | NS | 1.033(0.867-1.232) |

SNP, single-nucleotide polymorphism; VKH, Vogt-Koyanagi-Harada; OR, odds ratio; NS, not significant;95 % CI, 95 % confidence interval; Pc, Bonferroni corrected p value
